# Supplementary material for: Providing context: Extracting non-linear and dynamic temporal motifs from brain activity
Source: bioRxiv. 2024 Jun 27:2024.06.27.600937. Preprint. [Version 1] doi: 10.1101/2024.06.27.600937 (PMC11230350; doi:10.1101/2024.06.27.600937)
Supplement: Supplement 1 [file NIHPP2024.06.27.600937v1-supplement-1.pdf]

bioRxiv preprint doi: https://doi.org/10.1101/2024.06.27.600937; this version posted June 27, 2024. The copyright holder for this preprint (which was not certified by peer review) is the author/funder, who has granted bioRxiv a license to display the preprint in perpetuity. It is made available under aCC-BY 4.0 International license.

Supporting information

1 Appendix A: Hyperparameter ranges

To select the best hyperparameter combination, we use Ray Tune [23], and Optuna search [24]. Specifically, we use an ASHA scheduler that minimizes the validation mean squared error loss, with a grace period of 50 epochs, 1 bracket, and a maximum number of scheduler epochs of 150. Then, when the best hyperparameter setting is selected, we train the model for another 850 epochs. We apply early stopping, with 50 epochs of patience, both during this last training phase and the scheduler training phase. To select new hyperparameter settings, we use the default Optuna Search [24], and the hyperparameter ranges presented in Tabel 2.

Hyperparameter	Num layers	Spatial hidden size	Temporal hidden size	Dropout	β	γ
Range	[3, 4, 5]	[128, 256]	[256, 512]	[0.0, 0.1, 0.2]	[1E-3, 1E-4]	[1E-4, 1E-5]

Table 2. The hyperparameter ranges that were optimized over.

bioRxiv preprint doi: https://doi.org/10.1101/2024.06.27.600937; this version posted June 27, 2024. The copyright holder for this preprint (which was not certified by peer review) is the author/funder, who has granted bioRxiv a license to display the preprint in perpetuity. It is made available under aCC-BY 4.0 International license.

2 Appendix B: Geometric manifold comparison visualization

To visually verify that our model's embedding space is not capturing wFNC features, we visualize our model's embedding space using the Jonker-Volgenant algorithm [33] in Figure 6. The figure is created by first embedding each of the windows using our LS=2,CS=2 model, the same model we used in Section Cluster analysis. Then, we used the Jonker-Volgenant algorithm to create from the locations of points in the embedding space, note each point corresponds to a window in the rs-fMRI signal. Lastly, we visualized each point/window as its wFNC, this allows us to see whether wFNC patterns are close together in our model's embedding space. If this is not the case, then our model is likely capturing complementary features that are important (see Section Window classification), and interesting (see Section Cluster analysis). In

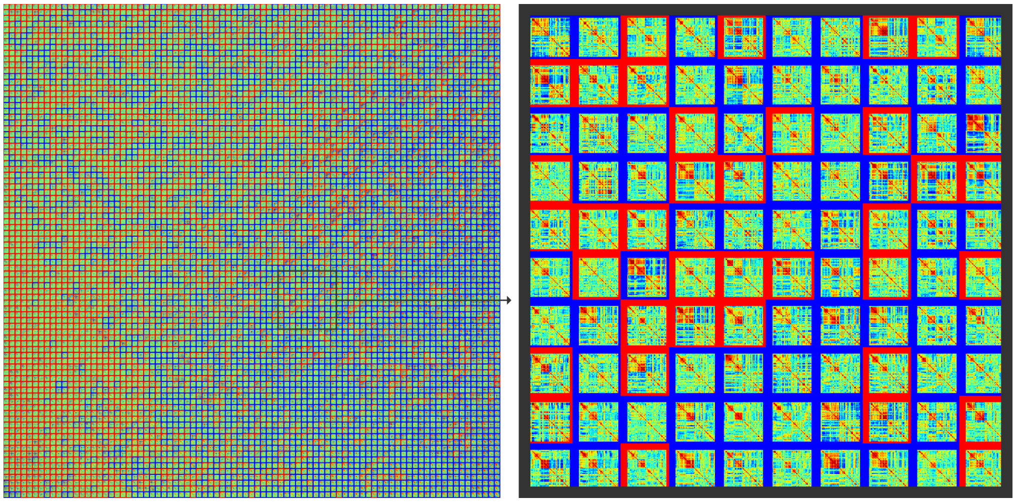

Fig 6. The left plot shows the points in our model's embedding space represented as wFNC matrices. Each wFNC's border is either red or blue; red corresponds to windows that are from schizophrenia patients and blue corresponds to windows from control subjects. The right side shows a zoomed-in version of the subplot on the left.

Figure 6, we can see that similar wFNCs are not per se close together in our model's embedding space. Since correlation is invariant to scaling, temporal permutation (if the permutation is the same for all inputs), and the addition of a constant, connectivity features from correlations are highly specific. Indeed, we can visually see that our model captures different features than wFNC does. Thus, our method can uncover novel yet complementary motifs for this schizophrenia population from rs-fMRI data.
